# Markedly improved disease control in Darier disease (*ATP2A2*-nonsyndromic epidermal differentiation disorder) with ustekinumab versus other biologics: a case series

**DOI:** 10.1093/skinhd/vzag072

**Published:** 2026-06-23

**Authors:** Anna Skarnvad Andersen, Elena Baez, Thomas Emmanuel, Kirsten Rønholt, Mette Sommerlund, Claus Johansen

**Affiliations:** Department of Dermatology, Aarhus University Hospital, Aarhus, Denmark; Department of Clinical Medicine, Aarhus University Hospital, Aarhus, Denmark; Department of Dermatology, Aarhus University Hospital, Aarhus, Denmark; Department of Clinical Medicine, Aarhus University Hospital, Aarhus, Denmark; Department of Dermatology, Aarhus University Hospital, Aarhus, Denmark; Department of Clinical Medicine, Aarhus University Hospital, Aarhus, Denmark; Department of Dermatology, Aarhus University Hospital, Aarhus, Denmark; Department of Clinical Medicine, Aarhus University Hospital, Aarhus, Denmark; Department of Dermatology, Aarhus University Hospital, Aarhus, Denmark; Department of Clinical Medicine, Aarhus University Hospital, Aarhus, Denmark; Department of Dermatology, Aarhus University Hospital, Aarhus, Denmark; Department of Clinical Medicine, Aarhus University Hospital, Aarhus, Denmark

## Abstract

In this case series, we report six patients with severe, treatment-refractory Darier disease, or dyskeratosis follicularis, also known as *ATP2A2*-nEDD (nonsyndromic epidermal differentiation disorder), treated with off-label biologic therapy, including dupilumab (*n* = 3), secukinumab followed by guselkumab (*n* = 1), and ustekinumab (*n* = 2). All patients had longstanding disease with recurrent flares, frequent infections and substantial impairment of quality of life, despite multiple conventional treatments. All biologic treatments were well tolerated, with no serious adverse events. Dupilumab consistently improved pruritus but had limited effects on skin lesions. Secukinumab and guselkumab provided only transient or partial disease control. In contrast, both patients treated with ustekinumab achieved pronounced and sustained clinical improvement, with markedly reduced disease severity and pruritus, and considerable improved quality of life. The pronounced and sustained responses observed with ustekinumab support its prioritization for future studies in Darier disease.

## Teaser

We report a case series of six patients with severe, treatment-refractory Darier disease treated off-label with biologic agents. Treatment responses varied, with marked improvement observed with ustekinumab, supporting further investigation of IL-12/23 inhibition in Darier disease.

What is already known about this topic?

- Recent case reports have described clinical improvements in patients with Darier disease, or dyskeratosis follicularis, also known as *ATP2A2*-nEDD (nonsyndromic epidermal differentiation disorder), treated with biologic therapies.
- Reported responses are variable and sometimes transient, highlighting uncertainty regarding optimal therapeutic targets and the durability of effect.

What does this study add?

- Our clinical observations highlight interleukin-12/-23 inhibition as a promising therapeutic strategy in patients with *ATP2A2*-nEDD, warranting prioritization in future studies.

Darier disease, also known as dyskeratosis follicularis, and recently renamed as *ATP2A2*-nEDD (nonsyndromic epidermal differentiation disorder), is a rare, autosomal dominant inherited skin disease with a chronic relapsing course. Common symptoms, such as pruritus, malodour, painful erosions and secondary infections, markedly impair patients’ quality of life.^1^ While no established disease-modifying therapy is currently available, emerging research suggests that targeted or immunomodulatory approaches may become relevant in the future, especially given the underlying immunological aspects of *ATP2A2*-nEDD.^2,3^ Available treatments include topical corticosteroids, systemic retinoids and antibiotics; however, they are often inadequate and limited by side effects.^4^ Recent case reports suggest that targeted immunomodulation may offer therapeutic benefits in patients with *ATP2A2*-nEDD, with improvements reported following T helper (Th)2- and Th17/Th1-targeting agents.^2,5^ Here, we present six patients with severe, treatment-­refractory *ATP2A2*-nEDD (Table 1) treated off-label with biologics (dupilumab, *n* = 3; secukinumab and subsequent guselkumab, *n* = 1; ustekinumab, *n* = 2), reflecting a strategy to target different inflammatory pathways due to the overlapping therapeutic responses observed.^2,5^ The biologic treatment regimens and clinical assessments are summarized in Table 2.

**Table 1:** Clinical characteristics of patients with Darier disease, also known as *ATP2A2*-nEDD (nonsyndromic epidermal differentiation disorder) included in this case series

Patient	Sex/age (years)/ethnicity	BMI (kg m^–2^)	Concurrent treatment	Previous treatment	Diagnosis confirmation
1	F/62/White	27.2	Topical: antiseptics, corticosteroids; systemic: retinoid (acitretin 25 mg daily)	Topical: antiseptics, antiperspirants, corticosteroids, corticosteroids/antiseptics; systemic: antibiotics, retinoids (acitretin, isotretinoin)	Genetically confirmed *ATP2A2* variant (ACMG class V)
2	F/31/White	30.1	Topical: corticosteroids, corticosteroids/antiseptics; systemic: antibiotics (dicloxacillin 1 g four times daily for 14 days, initiated at week 6), retinoid (isotretinoin 10 mg every third day; discontinued at week 12)	Topical: antiseptics, corticosteroids, corticosteroids/antiseptics, retinoids; systemic: antibiotics, antivirals, retinoid (isotretinoin), corticosteroid	Genetically confirmed *ATP2A2* variant (ACMG class V); histologically confirmed Darier disease
3	M/31/White	NA	Topical: corticosteroids, corticosteroids/antiseptics, calcineurin inhibitor; systemic: retinoid (acitretin 25 mg daily; increased to 50 mg daily at week 15)	Topical: antiseptics, keratolytics, corticosteroids, corticosteroids/antiseptics, corticosteroids/vitamin D analogue, calcineurin inhibitor; systemic: antibiotics, antivirals, retinoid (acitretin), methotrexate	Genetically confirmed *ATP2A2* variant (ACMG class IV); histologically confirmed Darier disease
4	M/37/White	35.7	Topical: corticosteroids, corticosteroids/antiseptics	Topical: antiseptics, corticosteroids, corticosteroids/antiseptics, calcineurin inhibitor; systemic: antibiotics, antivirals, retinoids (acitretin, isotretinoin)	Genetically confirmed *ATP2A2* variant (ACMG class IV); histologically confirmed Darier disease
5	M/52/White	24.2	Topical: antiseptics, corticosteroids; systemic: antibiotics (dicloxacillin 1 g four times daily for 3 days initiated at week 3; switched to intravenous cloxacillin 1 g four times daily during 9 days of hospitalization; discharged on dicloxacillin 1 g four times daily for 14 days), antiviral (valaciclovir 1 g three times daily during hospitalization; reduced to prophylactic dose 500 mg twice daily at discharge; discontinued at month 4), retinoid (acitretin 25 mg daily initiated during hospitalization at week 3; discontinued at week 17)	Topical: antiseptics, keratolytics, corticosteroids, corticosteroids/antiseptics; systemic: antibiotics, antivirals, retinoid (acitretin)	Histologically confirmed Darier disease
6	M/19/White	20.8	Topical: antiseptics, corticosteroids, calcineurin inhibitor; systemic: retinoid (acitretin 25 mg daily; tapered to 10 mg daily by week 33)	Topical: antiseptics, antiperspirants, keratolytics, corticosteroids, corticosteroids/antiseptics, corticosteroids/vitamin D analogue, retinoids, calcineurin inhibitor; systemic: antibiotics, retinoids (acitretin, isotretinoin)	Genetically confirmed *ATP2A2* variant (ACMG class IV); histologically confirmed Darier disease

Concurrent treatment refers to concomitant treatments administered during biologic therapy. ACMG, American College of Medical Genetics; BMI, body mass index; F, female; M, male; NA, not available.

**Table 2:** Clinical evaluation of pruritus was performed at each visit

Biologic treatment regimens				
Patient no.	Biologic agent	Mechanism of action	Dosage and schedule	Clinical visits (weeks)
1	Dupilumab (Dupixent^®^)	Anti-IL-4Rα-antibody inhibiting IL-4 and IL-13 signalling	300 mg every 2 weeks after initial loading dose of 600 mg	0, 6, 13 and 26
2			300 mg every 2 weeks after initial loading dose of 600 mg; 300 mg weekly from week 6	0, 6, 15 and 35
3			300 mg every 2 weeks after an initial loading dose of 600 mg	0, 7 and 15^a^
4	Secukinumab (Cosentyx^®^)	IL-17A inhibitor	300 mg once weekly for 5 weeks, then once monthly	0, 2, 4, 8 and 12
	Guselkumab (Tremfya^®^)	IL-23p19 inhibitor	100 mg at weeks 0 and 4, then every 8 weeks	0, 6, 12, 20, 29 and 37
5	Ustekinumab (Uzpruvo^®^)	IL-12/IL-23 p40 inhibitor	45 mg at weeks 0 and 4, then every 12 weeks	0^b^, 4, 9, 27 and 33
6				0^b^, 4, 8, 16 and 37
**Clinical evaluations**				
**Parameter**		**Details**		
Clinical examination		Vital signs, physical exams, adverse events and treatment effects were used to evaluate treatment efficacy, safety and tolerability		
Medical photography		Standardized full-body clinical photographs were obtained to monitor changes in skin lesions over time		
**Patient-reported outcome measures**				
DLQI^6^		A 10-item questionnaire, used to assess the impact of Darier disease, also known as *ATP2A2*-nEDD (nonsyndromic epidermal differentiation disorder), on patients’ quality of life over time. Each item is scored on a 4-point scale (0 = not at all/not relevant; 1 = a little; 2 = a lot; 3 = very much), yielding a total score from 0 to 30		
Pruritus-NRS, Pruritus-VRS, Pruritus-VAS		Pruritus severity was assessed using the following patient-reported scales:^7^ Pruritus-NRS ranging from 0 to 10 (0 = no itch; 10 = worst imaginable itch); Pruritus-VRS based on a five-category scale ranging from 0 to 4 (0 = no itch; 1 = low itch; 2 = moderate itch; 3 = severe itch; 4 = very severe itch); Pruritus-VAS consisting of a 10-cm line labelled 0–10 (0 = no itch; 10 = worst imaginable itch)		

DLQI, Dermatology Life Quality Index; IL, interleukin; NRS, numeric rating scale; VAS, visual analogue scale; VRS, verbal rating scale. ^a^No patient-reported outcome data were collected for patient 3. ^b^No patient-reported outcome data were collected at week 0 for patients 5 and 6.

## Case series

**Figure 1:**
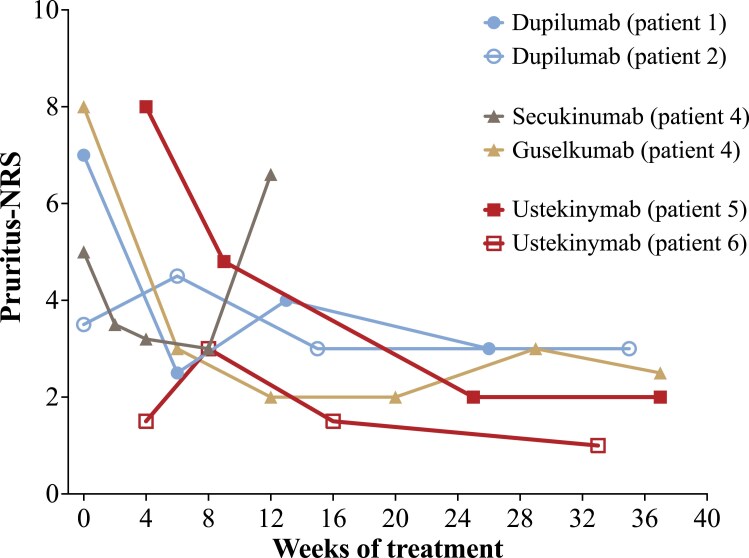
Changes in patient-reported pruritus intensity during biologic treatment, assessed by the Pruritus Numeric Rating Scale (Pruritus-NRS), ranging from 0 (no pruritus) to 10 (very severe pruritus). Patient 4 received sequential treatment with secukinumab followed by guselkumab. For patients 5 and 6, patient-reported outcomes are presented from week 4 onwards due to missing baseline data. No patient-reported outcome data were available for patient 3.

**Figure 2:**
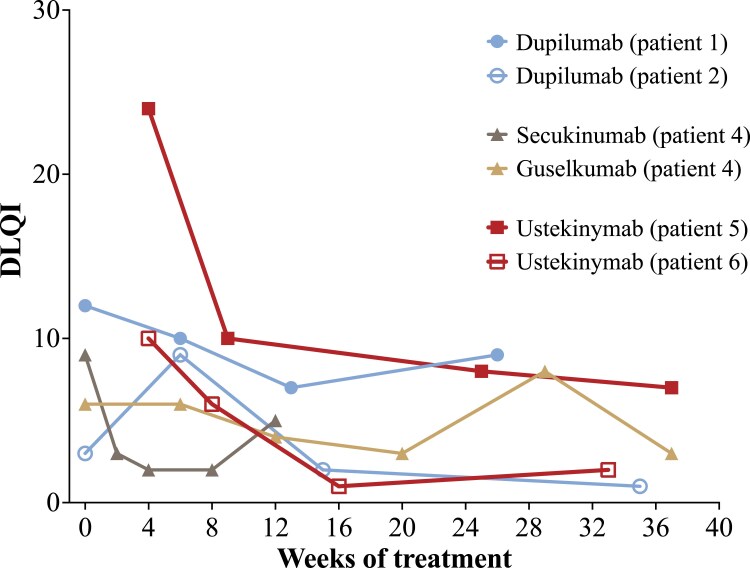
Changes in the impact of Darier disease, also known as *ATP2A2*-nEDD (nonsyndromic epidermal differentiation disorder), on quality of life during biologic treatment, assessed using the Dermatology Life Quality Index (DLQI), a 10-item patient-reported questionnaire with a score that ranges from 0 to 30 (higher scores indicate greater impairment). Patient 4 received sequential treatment with secukinumab followed by guselkumab. For patients 5 and 6, patient-reported outcomes are presented from week 4 onwards due to missing baseline data. No patient-reported outcome data were available for patient 3.

Three patients (patients 1–3) initiated dupilumab, an interleukin (IL)-4/IL-13 inhibitor, due to debilitating pruritus (600-mg loading dose, then 300 mg twice weekly). All had longstanding, severe disease with recurrent flares complicated by secondary *Staphylococcus aureus* infections. Despite ongoing topical therapy and systemic retinoid treatment, sustained disease control was not achieved. Concomitant systemic retinoids (patients 1 and 3, acitretin 25 mg daily; patient 2, isotretinoin 10 mg every third day) and topical therapy were continued during treatment with dupilumab. Patient 1, a 62-year-old woman, reported improved pruritus and quality of life, despite no objective changes in skin lesions during 6 months of treatment. Patient 2, a 31-year-old woman, developed a sun-induced flare at week 6, requiring 14-day oral antibiotic treatment and escalation to dupilumab 300 mg weekly. Disease activity subsequently stabilized, pruritus improved and isotretinoin was discontinued. After 8 months, she reported sustained effects with ­minimal need for topical treatment. Patient 3, a 31-year-old man, reported reduced pruritus, but as skin lesions worsened, despite escalating acitretin to 50 mg daily at week 15, dupilumab was discontinued after 6 months. Overall, dupilumab relieved pruritus and improved the patient’s quality of life (Figures 1, 2; patients 1 and 2) with little impact on cutaneous *ATP2A2*-nEDD lesions. The only adverse event was dry eyes, which was manageable with lubricating drops (patients 1 and 3).

A 37-year-old man (patient 4) initiated secukinumab (anti-IL-17A), guided by skin-biopsy analysis revealing fivefold higher *IL17A* and twofold higher *IL23A* mRNA expression in lesional skin compared with nonlesional skin and skin from healthy controls. Disease control remained inadequate with available standard therapies, including topical treatments and systemic retinoids. The patient reported disease exacerbations during periods of increased work-related stress, resulting in functional impairment and sick leave. Secukinumab 300 mg was administered once weekly in the first 5 weeks, followed by once monthly for a total of 12 weeks. Pruritus and quality of life initially improved but worsened by week 12 (Figures 1, 2). Skin lesions and excoriations likewise worsened, prompting treatment discontinuation at week 12. Subsequent guselkumab, an IL-23p19 inhibitor (100 mg at weeks 0 and 4, and then every 8 weeks), provided partial stabilization, yet gradual skin worsening led to discontinuation after 8 months. Despite skin deterioration, guselkumab offered more sustained itch relief than secukinumab (Figure 1). Quality of life paralleled pruritus severity (Figure 2).

**Figure 3:**
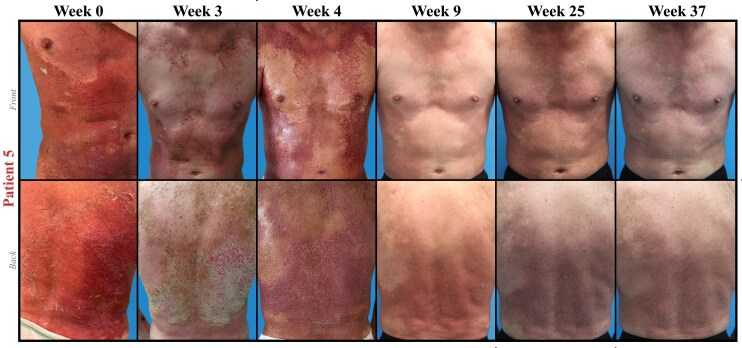
Clinical photographs of patient 5 during treatment with ustekinumab.

**Figure 4:**
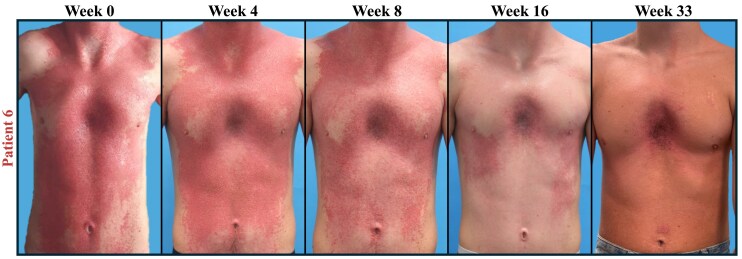
Clinical photographs of patient 6 during treatment with ustekinumab.

Inspired by a reported case of sustained remission with ustekinumab (anti-IL-12/IL-23p40) in *ATP2A2*-nEDD, including the absence of hospital readmissions,^8^ we initiated ustekinumab in two patients (patients 5 and 6) during hospitalization-requiring flares, in combination with intensified topical therapy. Both patients had a history of recurrent hospitalizations due to disease exacerbations and secondary infections despite ongoing systemic retinoid treatment and extensive topical therapy. Ustekinumab 45 mg was administered at weeks 0 and 4, and then every 12 weeks. Patient 5 (52-year-old man) had been taking acitretin 25 mg daily only intermittently in the months preceding admission, and systemic retinoid treatment was therefore not continued at ustekinumab initiation. In contrast, patient 6 (a 19-year-old man) was receiving acitretin 25 mg daily at the time of ustekinumab initiation, which was continued during treatment. Three weeks after ustekinumab initiation, patient 5 was readmitted with bacterial and herpes simplex infections, which were managed with intravenous antibiotics, acitretin 25 mg daily and topical treatment, while ustekinumab was continued. Despite this early complication, which was probably unrelated to ustekinumab, he achieved near-complete skin clearance within 9 weeks (Figure 3), enabling acitretin discontinuation after 4 months of ustekinumab. Disease remained controlled at week 37 (Figure 3). Patient 6 showed gradual disease improvement from week 8, with a marked reduction in severity by week 16 (Figure 4), allowing acitretin to be tapered from 25 to 10 mg daily by week 33. Both patients achieved unprecedented, sustained disease control, including meaningful improvements in pruritus and quality of life (Figures 1–4).

## Discussion

These six cases illustrate heterogeneous responses to biologics in *ATP2A2*-nEDD. Dupilumab mainly alleviated pruritus, consistent with a previous report,^9^ whereas others have reported more pronounced skin improvement with IL-4/IL-13 blockade.^10^ Similarly, variable and transient effects have been described with IL-17 and IL-23 inhibitors in *ATP2A2*-nEDD,^5,11^ consistent with our observations in patient 4 during secukinumab and guselkumab treatment. Such variability may reflect the fluctuating course of DD and/or patient-specific factors, including inflammatory profile, microbiome composition, genetic variant and clinical phenotype. In contrast, ustekinumab led to clear improvement in patients 5 and 6. Concomitant acitretin may have contributed synergistically, which has been previously proposed,^11^ yet both patients improved sufficiently to taper or ­discontinue acitretin, supporting an independent effect of ustekinumab. While placebo effects or nonspecific benefits from closer follow-up cannot be excluded, the extent and durability of effects in these patients with long-standing, severe disease, building on a previous report,^8^ highlight IL-12/IL-23 inhibition as a promising therapeutic strategy. Accordingly, patients 1 and 4 have recently initiated ustekinumab, patient 3 awaits initiation, whereas patient 2 remains satisfied with the antipruritic effects of dupilumab and continues this therapy.

Importantly, all biologics were well tolerated irrespective of outcome, supporting the safety and rationale for further evaluation in this patient group predisposed to secondary infections. Continued long-term follow-up will provide further insight into the use of biologics, particularly ustekinumab. Larger, prospective cohorts integrating molecular profiling are warranted to identify responsive subgroups and guide evidence-based management of this rare but burdensome disease.
